# The impact of university teachers’ transformational leadership on students’ social–emotional competence: the mediating role of teacher–student relationship and learning engagement with moderated by self-efficacy

**DOI:** 10.3389/fpsyg.2025.1657492

**Published:** 2025-10-23

**Authors:** Lin Lin, Chunying Wang

**Affiliations:** ^1^College of Chinese Studies and Foreign Languages, Yantai Nanshan University, Longkou, China; ^2^Department of Business Administration, Joongbu University, Daejeon, Republic of Korea

**Keywords:** transformational leadership, social–emotional competence, teacher–student relationship, learning engagement, self-efficacy

## Abstract

In the context of increasing emphasis on holistic student development in higher education, transformational leadership (TL) demonstrated by university faculty has been linked to positive student outcomes, including social–emotional competence (SEC). This study explored the associations between TL and students’ SEC by examining two potential mediating processes—the quality of teacher–student relationships (TSR) and students’ learning engagement (LE)—as well as the moderating role of self-efficacy (SE). Survey data were collected from 659 undergraduates at multiple universities in China. Using structural equation modeling (SEM) and moderated mediation analysis (PROCESS Model 7), the findings revealed that TL positively influenced students’ SEC through two parallel pathways: by strengthening TSR and enhancing LE. Notably, SE significantly moderated the relationship between TL and TSR, such that the indirect effect of TL on SEC via TSR was stronger among students with higher levels of SE. However, the SE-moderated path via LE was not significant. These results highlight the importance of both relational and motivational processes in leadership-informed pedagogy, and underscore how students’ psychological traits such as self-efficacy condition their responsiveness to instructional leadership. Although the cross-sectional design limits causal inference, this study provides initial empirical support for targeted leadership strategies that align with students’ individual resources to foster social–emotional development in higher education.

## 1 Introduction

In recent years, higher education institutions globally have shifted focus toward the students’ holistic development, realizing that mere academic success is no longer enough to equip them for the complexities of contemporary life, work, and society. Social–emotional competence (SEC), defined as the capacity to regulate emotions, foster healthy relationships, and make responsible decisions, has thus emerged as a key focus in educational research and policy (CASEL, 2020; OECD, 2021). That enables teenagers to navigate socially demanding contexts through effective adaptation and interpersonal growth, thereby serving as a cornerstone for their holistic development across cognitive, behavioral, and psychological domains. SEC is associated not only with academic achievement but also with psychological resilience, enhanced interpersonal communication, career adaptability, and greater life satisfaction (Schaufeli et al., 2002; Osher et al., 2016; Abrahams et al., 2019).

Previous studies have predominantly emphasized structured social–emotional learning (SEL) programs or psychological interventions specifically designed to enhance students’ SEC. Meta-analyses have confirmed that well-implemented SEL programs significantly improve students’ social behaviors, emotional regulation, and academic performance across developmental stages (Durlak et al., 2011; Taylor et al., 2017). However, less empirical attention has been paid to how the inherent features of educational environments—particularly teacher behaviors and leadership styles—contribute to students’ social–emotional development beyond formal curricula (Jones et al., 2013; Domitrovich et al., 2017).

Among various leadership frameworks, transformational leadership (TL)—which involves inspiring followers to transcend immediate self-interests for shared goals through idealized influence, inspirational motivation, intellectual stimulation, and individualized consideration (Bass and Avolio, 1995)—has gained recognition for its potential to foster deep, sustained personal and interpersonal growth (Bass, 1985; Burns, 2003). Transformational teaching has been associated with increased student engagement, improved emotional regulation, and stronger relational bonds in educational settings (Harms et al., 2018; Hofkens and Pianta, 2022).

Moreover, recent studies have emphasized the critical role of teachers’ TL behaviors in cultivating students’ academic and social–emotional outcomes. For example, Zadok et al. (2024) found that transformational classroom leadership enhances students’ motivation and collaborative learning behaviors, while Abuhassira et al. (2024) demonstrated that TL improves classroom climate and students’ self-concept. These findings resonate with evidence showing that self-efficacious students—those who perceive themselves as capable of achieving learning goals—are more likely to display persistence, positive emotional states, and prosocial behavior (Bandura, 1997; Usher and Pajares, 2008), all of which contribute to the development of SEC.

TL in teaching contributes to the creation of emotionally supportive classroom climates, which are essential for nurturing students’ SEC. By modeling empathy, encouragement, and responsiveness, transformational teachers help establish learning environments marked by psychological safety and emotional attunement (Hofkens and Pianta, 2022; Harms et al., 2018). Such environments naturally foster high-quality teacher–student relationships (TSR), characterized by mutual respect, trust, and emotional closeness. These relational dynamics are consistently identified as key predictors of student engagement, academic motivation, and psychological well-being (Roorda et al., 2011; Roorda et al., 2017).

Empirical studies have shown that students who experience supportive TSR demonstrate higher levels of emotional regulation, empathy, and cooperative behavior—core facets of social–emotional development (Hughes, 2011; Reyes et al., 2012). Furthermore, TSR not only facilitates emotional safety and behavioral adjustment but also serves as an indirect pathway through which leadership practices such as TL exert positive influence on students’ social–emotional outcomes (Davis, 2003; Pianta et al., 2012). Therefore, TL’ s capacity to shape relational and affective aspects of classroom life is central to its role in promoting holistic student development.

LE, defined as the degree of students’ cognitive, emotional, and behavioral involvement in learning activities, has been shown to mediate educational outcomes substantially. Highly engaged students display increased persistence, enthusiasm, and positive affect, directly facilitating their social–emotional growth. A longitudinal study of Chinese EFL learners confirmed that behavioral engagement fully mediated the effect of positive achievement emotions on academic performance, illustrating how students’ enthusiasm and persistence within learning activities impact achievement through their active engagement.(Feng and Hong, 2022). Teachers exhibiting transformational behaviors not only stimulate interest and intrinsic motivation but also promote deeper learning engagement through intellectual challenges and personalized attention, thus positively impacting students’ SEC (Zou et al., 2024).

Moreover, Bandura’s self-efficacy theory posits that individual differences in students’ beliefs about their capabilities significantly influence how they respond to external supports and motivational influences (Bandura, 1997). Recent studies have confirmed that SE plays a pivotal role in moderating the effectiveness of teacher-student interactions and learning engagement on students’ emotional and social development (Baños et al., 2023; Gao et al., 2023). Specifically, students with higher SE are more likely to leverage positive interpersonal relationships and supportive learning environments to enhance their emotional regulation, interpersonal competence, and overall SEC (Eriksen and Bru, 2023).

From this theoretical perspective, SE functions as a pivotal psychological resource that enhances the positive influence of TSR and LE on students’ SEC. Students with higher SE tend to display stronger emotional resilience, more adaptive coping strategies, and a greater capacity to internalize and respond to social and instructional support (Bandura, 1997; Komarraju and Nadler, 2013). These psychological assets enable them to better leverage the core dimensions of TL—such as individualized consideration, inspirational motivation, and intellectual stimulation—into enhanced social–emotional development (Choi and Kang, 2021).

Moreover, individuals with elevated levels of SE are generally more receptive to emotional input and are more likely to exhibit emotionally intelligent behaviors in interpersonal contexts (Hoyt and Blascovich, 2010). This perspective is supported by recent empirical evidence from Chinese higher education settings, where SE was found to significantly moderate the relationship between TL and emotional competence (Wang et al., 2024). These findings suggest that the effectiveness of transformational leadership is not uniformly experienced by all students, but rather depends on their motivational and psychological readiness to engage with such leadership practices (Ng and Feldman, 2010; Lian et al., 2012).

In response to these theoretical and empirical gaps, the present study aims to elucidate the underlying mechanism through which university teachers’ transformational leadership behaviors affect students’ SEC. Specifically, this research explores:

1. Direct effects of TL on students’ SEC.
2. Independent mediating roles of TSR and LE.
3. The moderating role of SE in the indirect effects of TL on SEC via TSR and LE.

This study aims to contribute to the transformational leadership in education and provides concrete recommendations for higher education practitioners seeking to enhance students’ comprehensive development through teachers’ leadership behaviors, relationship-building, learning engagement, and targeted interventions aimed at improving students’ SE.

The subsequent sections of the paper delineate the theoretical underpinnings, methodological approach, analytical results, and practical implications, enriching both academic and practitioner understandings of leadership-driven educational outcomes in higher education settings.

## 2 Theory and hypotheses development

### 2.1 The direct impact of transformational leadership on students’ social–emotional competence

TL, first introduced by Burns (1978) and further developed by Bass (1985), describes a leadership style that motivates followers to transcend personal interests in pursuit of collective, higher-order goals. Its core dimensions—idealized influence, inspirational motivation, intellectual stimulation, and individualized consideration—have been widely applied across organizational domains, including education (Leithwood and Jantzi, 2005).

In educational settings, transformational teaching is reflected in practices that address students’ individual needs, foster intellectual curiosity, promote intrinsic motivation, and support moral and ethical development (Bass, 1985; Osher et al., 2016). TL has been linked to the formation of shared visions, enhanced innovation, and stronger teacher–student relationships, which together foster collaborative and engaging learning environments (Sliwka et al., 2024; Cruz and Kim, 2023). A growing body of research also confirms its positive impact on teacher efficacy and student academic outcomes across different educational levels (Hallinger, 2003; Leithwood and Sun, 2012; Choi and Kang, 2021). In particular, TL has been associated with improvements in classroom climate, learning motivation, and students’ psychosocial development (Hofkens and Pianta, 2022).

Recent empirical studies provide further support for TL’ s cultural relevance in Chinese higher education. Zhao and Jiang (2025) found that TL predicted faculty career success via career adaptability among 605 university teachers in Gansu. Yu and Jang (2024) showed that dimensions such as intellectual stimulation and visionary communication enhanced teaching performance in Guangdong private universities. Zhang (2024) demonstrated that TL improved innovation performance in Beijing polytechnic institutions, both directly and through mediators such as innovation culture and motivation. Sun et al. (2025) further confirmed that TL enacted by university presidents enhanced faculty well-being through job crafting and teaching efficacy. Even in secondary education, Fang and Yu (2023) observed that group-oriented TL promoted organizational citizenship behaviors, particularly in high collectivist environments. Similarly, Wang and Berger (2010) reported that transformational behaviors among university instructors in China significantly enhanced student engagement and academic satisfaction.

Taken together, these findings highlight that while TL retains core effectiveness across cultural contexts, its application in China should be culturally attuned. Recognizing hierarchical structures, collective orientations, and relational expectations is essential to understanding how TL is interpreted and enacted in Chinese higher education. Therefore, this study adopts TL as a guiding theoretical framework while acknowledging the need for its contextual adaptation within the Chinese sociocultural and educational landscape.

SEC refers to individuals’ abilities to identify, manage, and regulate emotions effectively, build positive interpersonal relationships, and make responsible decisions (CASEL, 2020). Moreover, SEC is widely recognized as vital for students’ academic success, personal resilience, career development, and overall quality of life (OECD, 2018; OECD, 2021). Álamo and Falla (2023) found that significant linkages were identified between self-regulatory skills and motivation, social awareness and prosocial behavior, responsible decision-making capacities and moral emotions—all of which correlate with transformational leadership. While the importance of transformational leadership in education is widely acknowledged, direct empirical evidence connecting teacher’s leadership practices to students’ psychosocial development remains notably scarce particularly within higher education contexts.

Given this theoretical grounding, we propose our first hypothesis:

*Hypothesis 1:* Transformational leadership will be a significant positive predictor of students’ social-emotional competence.

### 2.2 The mediating roles of teacher–student relationship and learning engagement

TL has been consistently shown to influence not only student outcomes directly but also indirectly through interpersonal and motivational processes (Leithwood and Jantzi, 2006; Hofkens and Pianta, 2022). Two such processes particularly relevant in higher education contexts are TSR and LE.

#### 2.2.1 Teacher–student relationship as a mediator

The teacher–student relationship reflects the emotional, cognitive, and behavioral quality of interactions between students and instructors (Roorda et al., 2011). In transformational classrooms, teachers show empathy, provide individual support, and build trust, all of which are central to developing high-quality TSR (Davis, 2003; Quin, 2017). Prior research has found that such relationships promote students’ sense of belonging, emotional security, and openness to learning (Wang and Eccles, 2013), which are foundational to the development of SEC.

In Chinese contexts, where teacher authority and relational harmony are culturally emphasized, TSR plays a particularly important role in shaping students’ psychological and emotional development (Gu and Schweisfurth, 2015). Hence, we propose:

*Hypothesis 2a:* Teacher–student relationship mediates the relationship between transformational leadership and students’ social-emotional competence.

#### 2.2.2 Learning engagement as a mediator

LE refers to students’ cognitive, emotional, and behavioral involvement in academic activities (Fredricks et al., 2004). Transformational teachers promote engagement by fostering autonomy, communicating high expectations, and stimulating students’ interest (Hofkens and Pianta, 2022). Engaged learners are more likely to develop perseverance, emotional regulation, and interpersonal skills—all of which are essential to SEC (Li and Lerner, 2011; Seli et al., 2016).

Empirical studies have demonstrated that TL is associated with higher engagement across cultural contexts, including China. Thus:

*Hypothesis 2b:* Learning engagement mediates the relationship between transformational leadership and students’ social emotional competence.

### 2.3 Theoretical rationale for the parallel mediation model

Prior research indicates that TSR can influence students’ LE, suggesting the possibility of a serial mediation pathway (e.g., Pianta et al., 2012). However, theoretical perspectives in higher education contexts also support the view that TSR and LE may operate as distinct yet complementary mechanisms through which TL fosters students’ SEC. In university settings, the increased autonomy of learners and the differentiated nature of classroom interactions may allow relational and motivational processes to develop independently rather than sequentially.

Transformational leadership theory (Bass, 1998) posits that TL can activate multiple follower outcomes simultaneously via distinct psychological mechanisms. The developmental model of school leadership (Hallinger, 2011) similarly emphasizes that relational (e.g., TSR) and motivational (e.g., LE) pathways can function concurrently to shape student development. Moreover, the Social and Emotional Learning (SEL) framework (CASEL, 2020) underscores the importance of both supportive relationships and active learning engagement as foundational contexts for SEC growth.

Taken together, these theoretical perspectives justify conceptualizing TSR and LE as parallel mediators in the TL–SEC relationship, reflecting two interrelated but independent routes through which transformational leadership can contribute to students’ SEC development.

### 2.4 The moderating role of self-efficacy

Self-efficacy (SE), a central construct in Bandura’s social cognitive theory (Bandura, 1997), reflects an individual’s belief in their capacity to achieve desired outcomes through their own actions. In educational contexts, SE operates not only as a motivational driver but also as a perceptual filter that shapes how students interpret and respond to external influences, including leadership behaviors. Students with high SE typically demonstrate greater motivation, persistence, adaptive learning strategies, and resilience when facing challenges (Schunk and DiBenedetto, 2016).

TL enhances TSR by fostering trust, respect, and open communication (Bass, 1998). However, students differ in the degree to which they perceive and internalize such relational cues. According to the differential susceptibility hypothesis (Belsky and Pluess, 2009), individuals’ personal resources can moderate their responsiveness to environmental inputs. SE represents one such resource: high-SE students tend to interpret teacher support as an opportunity to collaborate, seek feedback, and engage in mutual trust-building (Komarraju and Nadler, 2013), thereby strengthening TSR.

Empirical studies corroborate this moderating mechanism. Lian et al. (2012) found that high-SE individuals are more likely to view leaders’ behaviors as empowering, which promotes positive relational exchanges. Ng and Feldman (2010) similarly reported that SE amplifies the association between supportive leadership and interpersonal outcomes, as confident individuals are more proactive in initiating and sustaining high-quality relationships. In academic settings, Roorda et al. (2011) demonstrated that students with higher SE show greater emotional attunement and responsiveness to teachers’ relational gestures, leading to more positive TSR.

From a resource-based perspective (Hobfoll, 2011), SE can be conceptualized as a personal capital that enables students to leverage the social and emotional resources offered by transformational leaders. High-SE students are more inclined to reciprocate leadership support with trust and engagement, while low-SE students may underutilize or even disregard these relational opportunities. Therefore, SE is expected to moderate the TL–TSR pathway, strengthening the positive impact of TL on TSR when students possess high levels of efficacy.

*Hypothesis 3:* Self-efficacy moderates the relationship between transformational leadership and teacher–student relationship

In summary, this study systematically investigates the intricate mechanisms linking TL behaviors of university teachers to students’ SEC. Our conceptual model integrates direct, mediating, and moderating pathways to fully explain the mechanisms and boundary conditions of TL’s effects on SEC in higher education, advancing both leadership and student development research.

## 3 Methods

### 3.1 Participants and procedure

This study employed a quantitative, cross-sectional survey design. A total of 659 college students were recruited from multiple universities across several provinces in China using a randomized cluster sampling approach, ensuring broad representation across different academic disciplines and institutional contexts. Participants were eligible if they were full-time students enrolled at their respective institutions during the period of data collection.

Prior to data collection, this study was conducted in accordance with the Academic Ethics Norms and Measures for Handling Academic Misconduct of the first author’s University. The research protocol was reviewed by the university, which determined that the anonymous survey and analysis of non-sensitive data involved minimal risk to participants. Under the university’s institutional policy, such low-risk studies are exempt from formal ethics committee approval; therefore, no ethics approval ID was issued. We have also specified in the Ethics Statement that all participants were informed about the study purpose, the voluntary nature of participation, and the anonymity of responses before beginning the survey. Completion of the questionnaire was taken as informed consent, and no personally identifiable information was collected. The survey was administered online during regular class hours under standardized conditions to minimize external distractions and ensure data consistency. Completing the questionnaire took approximately 15–20 min per participant. The final sample included 342 female students (51.9%) and 317 male students (48.1%), with ages ranging from 17 to 22 years (M = 19.63, SD = 1.31).

### 3.2 Measurement instruments

Validated psychometric instruments were employed to measure all constructs involved: TL, TSR, LE, SEC, and SE. All measures were adapted from previously validated scales and translated into Chinese through a rigorous forward-backward translation procedure to ensure linguistic and cultural appropriateness.

#### 3.2.1 Transformational leadership (TL)

TL behaviors were assessed using the Chinese-adapted version of the Multifactor Leadership Questionnaire (MLQ), originally developed by Bass and Avolio (1995) and subsequently modified for the educational context by Qian and Lei (2010). The instrument comprises four conceptually distinct yet empirically related dimensions: individualized consideration, intellectual stimulation, inspirational motivation, and idealized influence. All items were rated on a 5-point Likert scale ranging from 1 (strongly disagree) to 5 (strongly agree).

To illustrate how the construct was operationalized, representative items for each dimension include:

- “My teacher encourages me to think about problems in new ways” (intellectual stimulation),
- “My teacher serves as a role model for me” (idealized influence),
- “My teacher expresses confidence that I can succeed” (inspirational motivation), and
- “My teacher considers my individual needs and abilities” (individualized consideration).

Although the MLQ is multidimensional in nature, we followed previous research practices (e.g., Judge and Piccolo, 2004; Podsakoff et al., 1990) by aggregating the subscale scores to form a composite measure of overall transformational leadership. This approach is commonly adopted when the research objective is to examine the general impact of transformational leadership rather than to disentangle dimension-specific effects. Moreover, the use of the composite score is supported by strong internal consistency (CR = 0.855; AVE = 0.596) and satisfactory discriminant validity with other constructs.

#### 3.2.2 Teacher–student relationship (TSR)

TSR was measured using a modified version of the Teacher–Student Relationship Scale originally developed by Pianta (1994), adapted to Chinese educational settings by Wang et al. (2001) and Zou et al. (2007). To ensure contextual relevance for university students, several items were reworded to reflect adult learner–instructor interactions while preserving the original constructs. The adaptation process involved expert review and pilot testing to confirm semantic clarity and cultural appropriateness.

The final version comprised two dimensions: closeness (e.g., “I feel comfortable talking to my teacher”) and conflict (e.g., “My teacher and I often misunderstand each other”), measured using 6 items on a 5-point Likert scale (1 = strongly disagree to 5 = strongly agree). Reliability and validity were satisfactory, with composite reliability (CR) = 0.957 and average variance extracted (AVE) = 0.792, supporting the scale’s internal consistency and construct validity in the higher education context.

#### 3.2.3 Learning engagement (LE)

LE was assessed using the Chinese version of the Learning Engagement Scale adapted by Fang et al. (2008), which retains 8 items to measure students’ cognitive, emotional, and behavioral involvement in academic activities. Although originally developed within the work engagement domain, the scale is based on the Utrecht Work Engagement Scale–Student version (UWES-S; Schaufeli et al., 2002), where “study” is conceptualized as the academic counterpart to “work.” Empirical studies have supported its applicability in university contexts and demonstrated its cross-cultural robustness. The items capture students’ positive psychological states toward learning—for instance, “I am enthusiastic about my studies” (dedication), and “I feel happy when I am studying intensively” (absorption). Responses were rated on a 5-point Likert scale from 1 (strongly disagree) to 5 (strongly agree). The scale demonstrated strong psychometric properties in the present study (Cronbach’s *α* = 0.88; CR = 0.935; AVE = 0.652), supporting its reliability and convergent validity.

#### 3.2.4 Social–emotional competence (SEC)

The SEC scale utilized in this study was adapted from the five-dimensional framework proposed by the Collaborative for Academic, Social, and Emotional Learning (CASEL, 2020), encompassing self-awareness, self-management, social awareness, relationship skills, and responsible decision-making. The 25-item scale was originally developed by scholars at the National Institute of Education, Nanyang Technological University, Singapore (Zhou and Ee, 2012), and has demonstrated strong psychometric properties in measuring students’ social–emotional competencies. In the present study, all items were rated on a 5-point Likert scale ranging from 1 (strongly disagree) to 5 (strongly agree). The adapted scale exhibited excellent internal consistency and convergent validity (CR: 0.936; AVE: 0.722), confirming its suitability for assessing university students’ social–emotional competence in the Chinese higher education context.

#### 3.2.5 Self-efficacy (SE)

SE, used as a moderating variable in this study, was assessed by a Chinese version of the General Self-Efficacy Scale (GSES), originally developed by Schwarzer and Jerusalem (1995) and translated into Chinese by Wang et al. (2001), which focusing on students’ confidence in managing learning tasks and overcoming challenges. Responses ranged from 1 (not at all true) to 5 (exactly true). Reliability analysis confirmed high internal consistency and robust validity, with Cronbach’s alpha values exceeding 0.90 in previous validation studies.

### 3.3 Statistical analysis

To ensure the robustness of the findings, we employed two complementary analytical approaches: Hayes’ PROCESS macro (Model 4) in SPSS 27.0 and structural equation modeling (SEM) using AMOS 26.0.

The PROCESS analysis was based on observed variables and estimated both direct and indirect effects of TL on SEC through the mediators TSR and LE. Bias-corrected 95% confidence intervals (CIs) were generated via 5,000 bootstrap resamples.

SEM, by contrast, modeled TL, TSR, LE, and SEC as latent variables with multiple indicators, thereby controlling for measurement error and providing an assessment of model fit. The maximum likelihood estimation method was used, and model fit was evaluated using standard indices, including the chi-square/df ratio, Comparative Fit Index (CFI), Tucker–Lewis Index (TLI), Root Mean Square Error of Approximation (RMSEA), and Goodness-of-Fit Index (GFI).

Both analyses tested the same hypothesized mediation model, enabling cross-validation of results under differing assumptions about measurement error and construct representation. The inclusion of SEM offers unique advantages: simultaneous estimation of measurement and structural models, explicit handling of measurement error, and evaluation of overall model adequacy—features not available in PROCESS.

## 4 Results

### 4.1 Descriptive statistics and correlations

Descriptive statistics for all measured variables are presented in Table 1. The mean scores ranged from 3.33 to 4.10, suggesting that participants generally reported moderate to high levels across constructs. Standard deviations ranged from 0.837 to 1.057, indicating acceptable variability.

**Table 1:** Descriptive statistics and correlations (*N* = 659).

Variable	Mean	SD	TL	SEC	TSR	SE	LE
TL	3.64	0.75	–				
SEC	3.84	0.79	0.417***	–			
TSR	3.97	0.69	0.503***	0.484***	–		
SE	3.82	0.76	0.410***	0.875***	0.476***	–	
LE	3.61	0.86	0.362***	0.763***	0.412***	0.801***	–

TL, Transformational Leadership; SEC, Social–Emotional Competence; TSR, Teacher–Student Relationship; SE, Self-Efficacy; LE, Learning Engagement. ***p < 0.001 (two-tailed).

To assess the distributional properties of the data, skewness and kurtosis statistics were calculated. Skewness values ranged from −0.802 to 0.026, and kurtosis values ranged from −0.811 to 0.218. These values fall within the recommended thresholds for approximate normality (|skewness| < 2; |kurtosis| < 7), suggesting that the data distribution did not significantly deviate from normality (Kline, 2016; West et al., 1995).

As a precaution against any minor violations of normality, bootstrapping procedures were applied in subsequent inferential analyses to ensure robust estimates (Hayes, 2018).

TL was positively correlated with all outcome variables: SEC (*r* = 0.417, *p* < 0.001), TSR (*r* = 0.503, *p* < 0.001), SE (*r* = 0.410, *p* < 0.001), and LE (*r* = 0.362, *p* < 0.001). The results indicate that students’ stronger perceptions of faculty transformational leadership correlate with improved outcomes spanning emotional, relational, motivational, and behavioral dimensions.

Notably, SEC was strongly correlated with SE (*r* = 0.875, *p* < 0.001) and LE (*r* = 0.763, *p* < 0.001), indicating that students with higher SE and stronger LE are more likely to report greater social–emotional competence. Additionally, TSR was significantly associated with both SE (*r* = 0.476, *p* < 0.001) and LE (*r* = 0.412, *p* < 0.001), highlighting the importance of relational closeness with teachers for fostering internal and behavioral engagement.

All correlation coefficients were statistically significant at the 0.01 level (two-tailed), supporting the theoretical assumptions regarding the interconnectedness of teachers’ leadership, motivation, and emotional development. These significant associations also provide empirical support for proceeding with the structural equation modeling to test the proposed mediation and moderation mechanisms.

### 4.2 Reliability and construct validity

To evaluate the psychometric properties of the scales, we assessed internal consistency reliability, convergent validity, and discriminant validity (Tables 2, 3). All constructs demonstrated high internal consistency, with Cronbach’s alpha values ranging from 0.901 to 0.969 across subscales. The overall scale reliability was excellent (*α* = 0.942) based on the total 34 items, meeting the standard of α > 0.70 (Nunnally and Bernstein, 1994).

**Table 2:** Discriminant validity, convergent validity, and inter-construct correlations.

Construct	AVE	√AVE	CR	TL	TSR	LE	SEC
TL	0.596	0.772	0.855	–	0.548	0.503	0.467
TSR	0.792	0.890	0.957	0.548	–	0.623	0.587
LE	0.652	0.807	0.935	0.503	0.623	–	0.665
SEC	0.722	0.850	0.936	0.467	0.587	0.665	–

**Table 3:** Structural path estimates for the hypothesized model.

Path	Estimate	S.E.	C.R.	*p*
Teacher–Student Relationship (TSR) ← Transformational Leadership (TL)	0.425	0.047	9.781	***
Learning Engagement (LE) ← Transformational Leadership (TL)	0.298	0.045	6.945	***
Socio-Emotional Competence (SEC) ← Teacher–Student Relationship (TSR)	0.360	0.042	9.061	***
Socio-Emotional Competence (SEC) ← Learning Engagement (LE)	0.338	0.039	9.219	***
Socio-Emotional Competence (SEC) ← Transformational Leadership (TL)	0.195	0.047	4.710	***

***Indicates *p* < 0.001.

Composite reliability (CR) values for each latent variable exceeded the recommended threshold of 0.70, and average variance extracted (AVE) values were all above 0.50, indicating satisfactory convergent validity (Hair et al., 2019; Kline, 2016).

Discriminant validity was confirmed using the Fornell-Larcker criterion: the square root of each construct’s AVE exceeded its inter-construct correlations (Fornell and Larcker, 1981; Schreiber et al., 2006), suggesting that all constructs were empirically distinct.

### 4.3 Multicollinearity and common method bias

Multicollinearity was assessed using variance inflation factors (VIF), which all fell within the acceptable range (VIF < 5), further supporting the robustness of the model (Hair et al., 2019). The highest VIF was 1.456 (TSR), well below the critical threshold of 5, indicating no concerning multicollinearity issues.

To address potential common method bias, Harman’s single-factor test was performed. The unrotated factor solution revealed that the first factor accounted for 44.2% of the total variance, below the 50% threshold, suggesting that common method bias was not a serious concern in this study (Podsakoff et al., 2003).

### 4.4 Mediation effects analysis

To test the mediating roles of TSR and LE in the relationship between TL and students’ SEC, a parallel mediation model (PROCESS Model 4) was conducted using a sample of 659 university students (Table 4).

**Table 4:** Model summary from PROCESS regression (*N* = 659).

Model	R	*R*^2^	Adj. *R*^2^	SE estimate	*R*^2^ Change	F Change	df1	df2	Durbin–Watson
1	0.790	0.624	0.622	0.486	0.624	362.25	3	655	2.049

The model demonstrated strong explanatory power, accounting for 62.4% of the variance in SEC (R^2^ = 0.624), with a significant F-test result (*F* = 362.250, *p* < 0.001). The Durbin–Watson statistic was 2.049, indicating no serious autocorrelation. Regression coefficients (Table 5) showed that TL significantly predicted SEC directly (*β* = 0.095, *p* = 0.001), while both TSR (*β* = 0.164, *p* < 0.001) and LE (*β* = 0.662, *p* < 0.001) were also significant predictors.

**Table 5:** Regression coefficients predicting SEC from TL, TSR, and LE.

Predictor	B	SE	Beta	*t*	*p*	Tolerance	VIF
TL	0.099	0.030	0.095	3.356	0.001	0.718	1.392
TSR	0.187	0.033	0.164	5.675	<0.001	0.687	1.456
LE	0.604	0.024	0.662	24.670	<0.001	0.798	1.252

The total effect of transformational leadership (TL) on students’ social–emotional competence (SEC) was positive and statistically significant, *β* = 0.436, SE = 0.037, t = 11.75, *p* < 0.001, 95% CI [0.363, 0.509]. When controlling for the mediators—teacher–student relationship (TSR) and learning engagement (LE)—the direct effect remained significant but was reduced in magnitude, *β* = 0.099, SE = 0.030, t = 3.356, *p* < 0.001, 95% CI [0.041, 0.157], indicating partial mediation.

**Figure 1:**
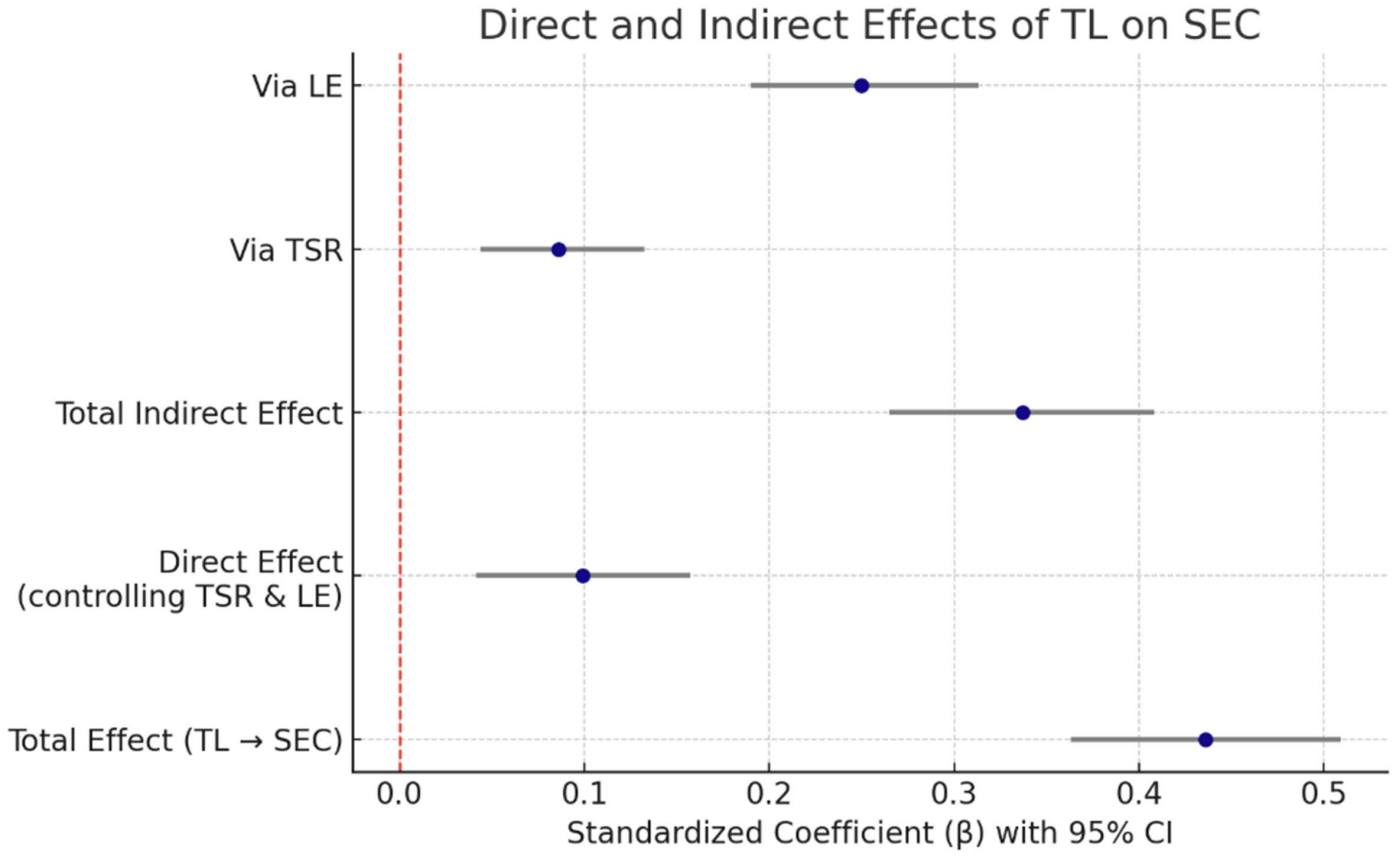
Forest plot: direct and indirect effects of TL on SEC.

The total indirect effect of TL on SEC through TSR and LE combined was statistically significant, *β* = 0.337, BootSE = 0.037, 95% CI [0.265, 0.408]. Examination of individual mediation pathways revealed that the indirect effect via TSR alone was significant, *β* = 0.086, BootSE = 0.023, 95% CI [0.044, 0.133], suggesting that TL enhances SEC in part by improving the quality of teacher–student relationships. The indirect effect via LE alone was also significant and comparatively stronger, *β* = 0.250, BootSE = 0.031, 95% CI [0.190, 0.313], indicating that TL’s influence on SEC is largely channeled through students’ engagement in learning activities (see Table 6, Figure 1).

**Table 6:** Direct and indirect effects of TL on SEC.

Effect type	*β*	SE/BootSE	*t*	*p*	95% CI Lower	95% CI Upper
Total effect (TL → SEC)	0.436	0.037	11.750	<0.001	0.363	0.509
Direct effect (controlling TSR & LE)	0.099	0.030	3.360	0.000	0.041	0.157
Total indirect effect	0.337	0.037	—	—	0.265	0.408
Indirect via TSR	0.086	0.023	—	—	0.044	0.133
Indirect via LE	0.250	0.031	—	—	0.190	0.313

BootSE, bootstrap standard error based on 5,000 resamples; CI, confidence interval; TSR, teacher–student relationship; LE, learning engagement; SEC, social–emotional competence.

### 4.5 Structural equation model

#### 4.5.1 SEM structural path results

Given the excellent model fit (see Table 7), the structural path analysis revealed that TL exerted significant positive effects on TSR (*β* = 0.425(0.43), *p* < 0.001) and LE (*β* = 0.300, *p* < 0.001). Both mediators independently transmitted the effect of TL to students’ SEC. Specifically, TSR significantly predicted SEC (*β* = 0.360, *p* < 0.001), and LE also significantly predicted SEC (*β* = 0.338(0.34), *p* < 0.001). TL further maintained a direct positive effect on SEC (*β* = 0.195(0.20), *p* < 0.001), indicating partial mediation.

**Table 7:** Model fit indices for the structural equation model.

Fit index type	Index	Value	Interpretation
Absolute fit indices			
	CMIN (χ^2^)	342.151	Significant, as *p* < 0.001
	DF	129	Degrees of freedom
	χ^2^/df (CMIN/DF)	2.652	Acceptable (between 1–3)
	RMR	0.026	Acceptable (closer to 0 is better)
	GFI	0.943	Good (≥ 0.90)
	AGFI	0.924	Good (≥ 0.90)
	PGFI	0.711	Acceptable (> 0.50)
Incremental fit indices			
	NFI	0.964	Excellent (≥ 0.95)
	RFI	0.958	Excellent (≥ 0.95)
	IFI	0.977	Excellent (≥ 0.95)
	TLI	0.973	Excellent (≥ 0.95)
	CFI	0.977	Excellent (≥ 0.95)
Parsimony fit indices			
	PRATIO	0.843	Indicates good model parsimony
	PNFI	0.813	Acceptable (≥ 0.50)
	PCFI	0.824	Acceptable (≥ 0.50)
Error approximation			
	RMSEA	0.050	Close fit (≤ 0.06)
	90% CI for RMSEA	[0.044, 0.057]	Indicates stable estimate

**Figure 2:**
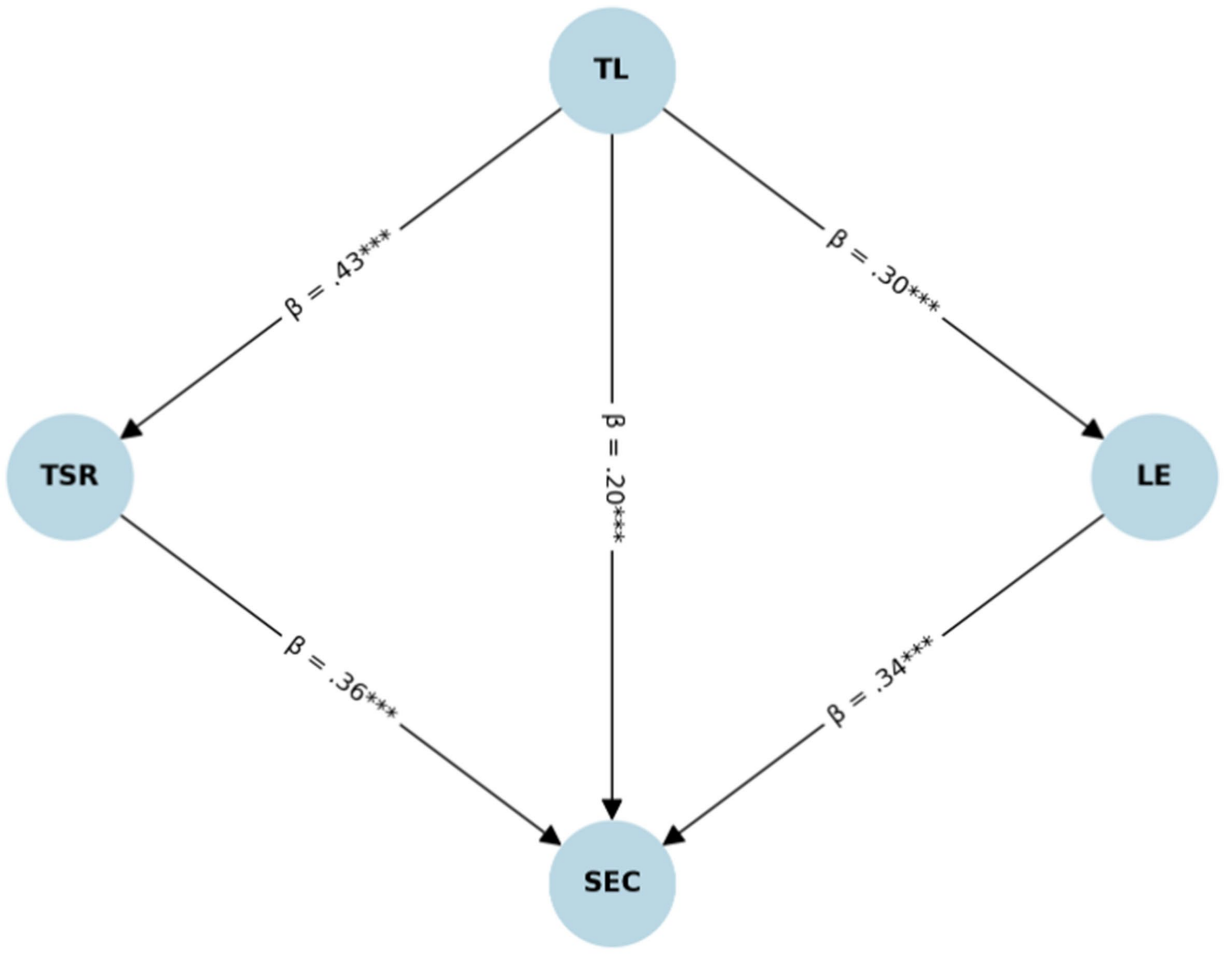
The structural equation model showing the effects of TL on students’ SEC, mediated by TSR and LE. Standardized path coefficients are reported; all paths are statistically significant at *p* < 0.001.

Bootstrapping with 5,000 resamples confirmed the significance of these mediation pathways. The indirect effect of TL on SEC via TSR was statistically significant (indirect effect = 0.174, 95% CI [0.129, 0.270]), as was the indirect effect via LE (indirect effect = 0.113, 95% CI [0.067, 0.159]). The total indirect effect was robust (0.510, 95% CI [0.404, 0.622]), underscoring the dual mediating roles of TSR and LE in linking TL to SEC (see Figure 2).

The dual analytical strategy adopted in this study—PROCESS and SEM—provides compelling evidence for the robustness of the mediation model linking transformational leadership to social–emotional competence through TSR and LE. While PROCESS offers a straightforward estimation of direct and indirect effects using observed variables, SEM adds unique value by modeling latent constructs, accounting for measurement error, and evaluating global model fit (Kline, 2016). The close alignment of results across both methods strengthens the validity of our conclusions and demonstrates that the observed effects are not artifacts of a particular statistical approach. By incorporating SEM, this study not only corroborates the PROCESS-based findings but also affirms the theoretical soundness and measurement integrity of the proposed model in the higher education context (see Table 8).

**Table 8:** Comparison of PROCESS and SEM estimates for mediation model.

Path	PROCESS *β* (BootSE)	95% CI	*p*	SEM Std. *β*	*p*
TL → SEC (Total effect)	0.436 (0.037)	[0.363, 0.509]	<0.001	0.44	<0.001
TL → SEC (Direct effect)	0.099 (0.030)	[0.041, 0.157]	0.001	0.10	0.002
TL → TSR → SEC	0.086 (0.023)	[0.044, 0.133]	–	0.09	<0.01
TL → LE → SEC	0.250 (0.031)	[0.190, 0.313]	–	0.25	<0.001

PROCESS estimates are based on observed variables; SEM estimates are based on latent variables. Both methods yielded identical significance patterns and highly similar effect sizes.

### 4.6 Moderated mediation analysis

#### 4.6.1 Moderation effects

We tested a moderated mediation model using PROCESS Model 7 with TL as the independent variable, SE as the moderator, TSR and LE as parallel mediators, and SEC as the outcome. The interaction between TL and SE significantly predicted TSR (*β* = 0.128, 95% CI [0.013, 0.247]), indicating that the effect of transformational leadership on teacher–student relationships was stronger for students with higher self-efficacy. However, the interaction effect on learning engagement was not significant (*β* = −0.018, 95% CI [−0.084, 0.051]), suggesting that self-efficacy did not significantly moderate the TL–LE link.

The final model showed significant indirect effects of TL on SEC through both TSR and LE. However, only the conditional indirect effect via TSR varied significantly with levels of SE. This implies that self-efficacy amplifies the impact of TL on SEC through enhanced teacher–student relationships, while its effect through learning engagement remains unchanged.

The results indicated a significant interaction between TL and SE on TSR (*β* = 0.128, *p* < 0.001), with the interaction accounting for a significant increase in explained variance (ΔR^2^ = 0.020, *F*(1, 655) = 15.97, *p* < 0.001). Specifically, conditional effects analysis revealed that the effect of TL on TSR increased as SE increased: the effect was significant and stronger at high levels of SE (Effect = 0.485, *p* < 0.001) compared to low levels (Effect = 0.197, *p* = 0.001). These results suggest that SE strengthens the positive association between TL and TSR.

In contrast, the interaction between TL and SE on LE was not significant (*β* = −0.018, *p* = 0.534), indicating that SE did not moderate the effect of TL on students’ learning engagement.

Furthermore, a moderated mediation analysis was conducted to assess whether the indirect effect of TL on SEC via TSR and LE varied as a function of SE. The index of moderated mediation for the TL → TSR → SEC pathway was significant (Index = 0.044, BootCI [0.006, 0.085]), confirming a significant moderated mediation. Conditional indirect effects showed that the indirect effect of TL on SEC via TSR increased at higher levels of SE, ranging from 0.067 at low SE to 0.165 at high SE. Conversely, the moderated mediation effect via LE was not significant (Index = −0.006, BootCI [−0.029, 0.020]).

**Figure 3:**
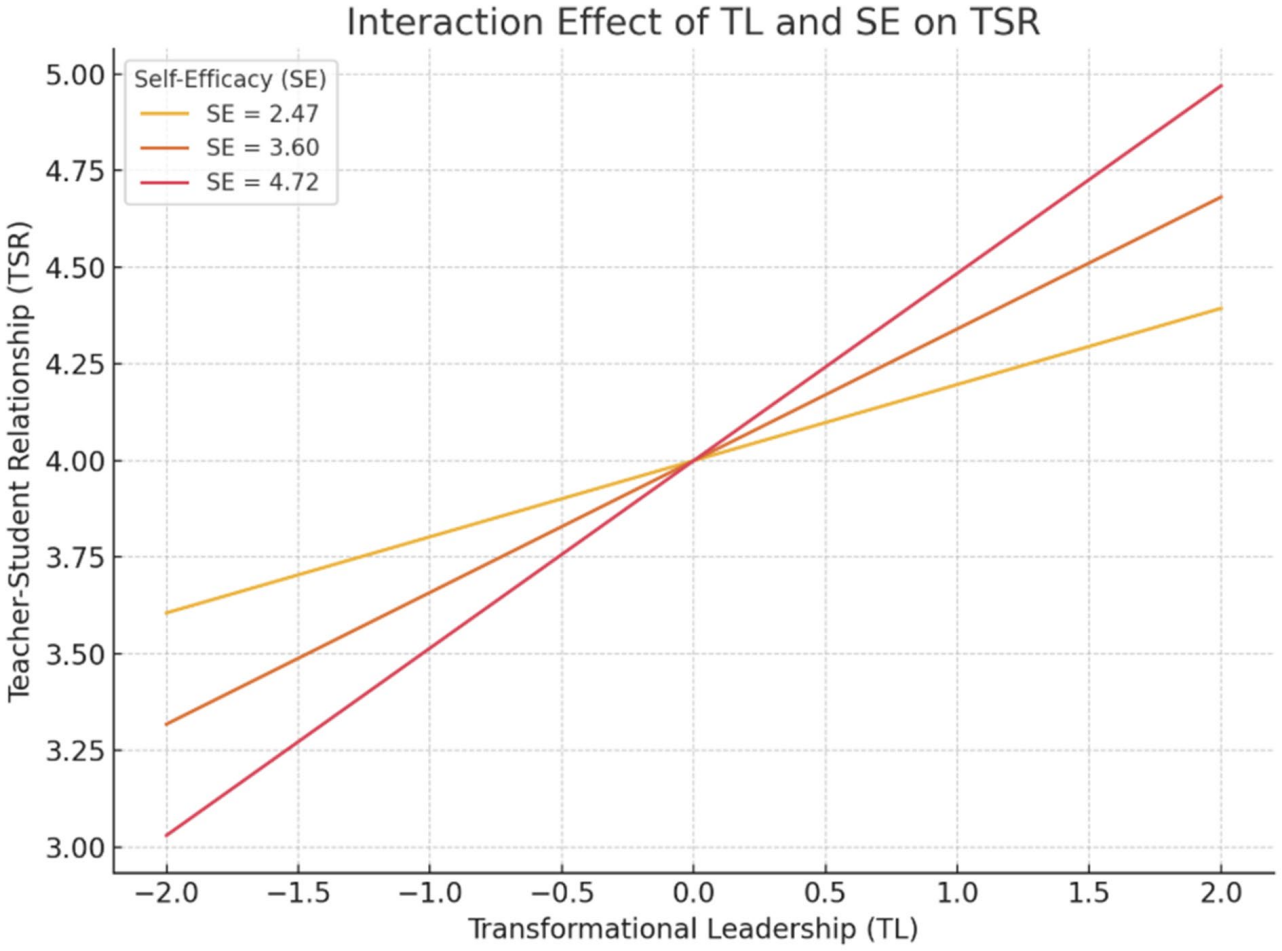
Interaction effect of transformational leadership and self-efficacy on teacher–student relationship.

In summary, SE moderated the first-stage path from TL to TSR but not to LE, and the indirect effect of TL on SEC via TSR—but not via LE—depended on students’ level of self-efficacy (see Figure 3).

**Figure 4:**
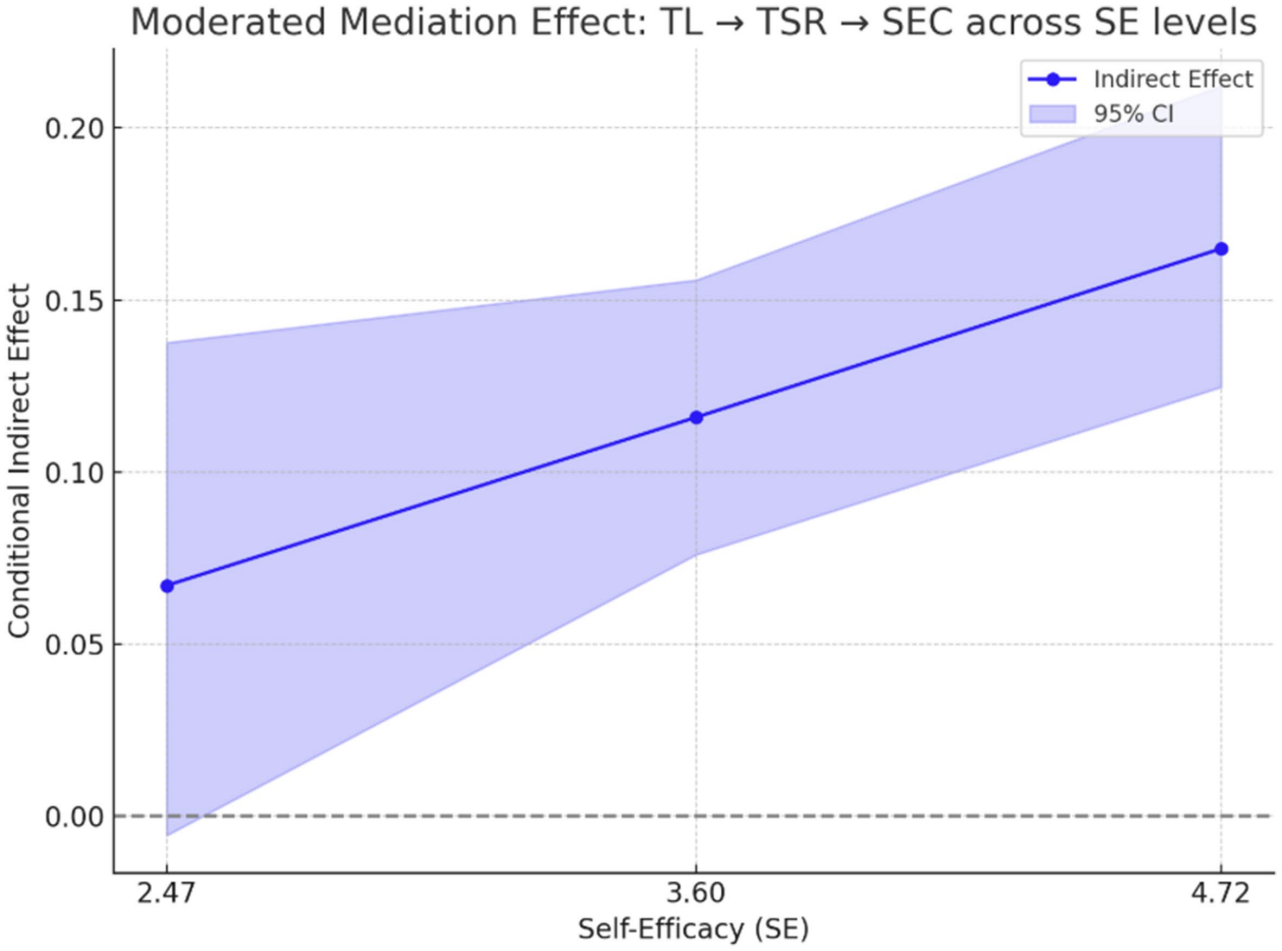
Conditional indirect effect of transformational leadership on social–emotional competence via teacher–student relationship across levels of self-efficacy.

Simple slope analysis illustrating the interaction between TL and SE in predicting TSR. The figure shows that TL has a stronger positive effect on TSR at higher levels of SE. Specifically, when SE is high (1 SD above the mean), the effect of TL on TSR is the strongest, while the effect is weaker at low SE levels. This interaction supports the hypothesis that SE moderates the TL → TSR relationship (see Figure 4).

Moderated mediation plot showing how the indirect effect of TL on SEC via TSR varies as a function of SE. The indirect effect becomes stronger and statistically significant at higher levels of SE. Shaded regions represent 95% bootstrap confidence intervals. These results provide evidence of a significant moderated mediation effect, indicating that SE enhances the indirect impact of TL on SEC through TSR.

## 5 Discussion

### 5.1 Summary of key findings

The present study aimed to explore how TL exhibited by university instructors affects students’ SEC, and whether this effect is mediated by TSR and LE, with SE acting as a moderator. The results provide nuanced insights into the conditional mechanisms by which TL facilitates students’ social–emotional development.

Consistent with prior research, TL was found to positively predict both TSR and LE, which in turn significantly contributed to SEC. These findings support the theoretical assumption that transformational leadership fosters emotionally supportive relationships and increases students’ behavioral and affective involvement in learning, both of which are crucial for social–emotional competence development.

### 5.2 Revisiting transformational leadership theory

Consistent with transformational leadership theory (Bass, 1985; Bass and Riggio, 2006), TL emerged as a significant predictor of SEC, both directly and indirectly. The significant indirect pathways via TSR and LE indicate that TL’s influence extends beyond the mere transmission of knowledge—it operates by cultivating trust, respect, and an emotionally supportive climate that promotes students’ socio-emotional growth. This aligns with prior work suggesting that individualized consideration and inspirational motivation can enhance learners’ capacity to manage emotions, establish positive relationships, and make responsible decisions (Harms et al., 2017). In the present study, instructors who displayed TL behaviors not only inspired students cognitively but also fostered the relational and motivational contexts that facilitate socio-emotional development.

### 5.3 Integration with the SEL framework

These findings also support the Social and Emotional Learning (SEL) framework proposed by the Collaborative for Academic, Social, and Emotional Learning (CASEL, 2020), which emphasizes that supportive adult–student relationships and active participation are critical in cultivating SEC. Although SEL research has traditionally focused on K–12 contexts (Durlak et al., 2011), our results demonstrate that the same mechanisms are relevant in higher education. The mediation findings suggest that TL behaviors can function as an instructional strategy that operationalizes SEL principles in adult learning environments, where SEC are often undervalued or assumed to be fully developed (Jones et al., 2021). This expands the SEL literature by positioning leadership style as a key antecedent in higher education.

### 5.4 Extension of social cognitive theory

From the perspective of social cognitive theory (Bandura, 1997, 2001), the moderating role of SE on the TL–TSR link indicates that students’ self-beliefs influence how they perceive and respond to leadership behaviors. High-SE students may be more likely to interpret TL behaviors as opportunities for growth and connection, thereby forming stronger relationships with instructors. This finding adds nuance to social cognitive theory by illustrating that leadership signals are not interpreted uniformly but are filtered through individual differences in perceived capability. Such differential responsiveness may be especially salient in university contexts, where students’ autonomy is higher and their engagement more self-directed compared to earlier educational stages (Schunk and DiBenedetto, 2020).

### 5.5 Reflection on the non-significant moderation effect of self-efficacy

Notably, SE did not significantly moderate the relationships between TL and LE, nor between TL and SEC. This suggests that while SE may enhance the relational benefits of TL—particularly in shaping TSR—it appears less influential in translating leadership behaviors into students’ engagement or direct socio-emotional outcomes. One possible explanation lies in the complex and multifaceted nature of engagement behaviors, which are shaped by a constellation of contextual factors such as peer dynamics, academic workload, instructional design, and institutional culture, potentially overshadowing the impact of individual efficacy beliefs (Fredricks et al., 2004; 2016; Kahu, 2013). Moreover, measurement limitations may have contributed to the non-significant moderation; general SE scales might lack sensitivity to domain-specific efficacy relevant to learning engagement tasks, thereby attenuating interaction effects (Chen et al., 2001).

Cultural and contextual factors are also critical considerations. In collectivist educational settings, student engagement often reflects social obligations, hierarchical teacher-student dynamics, and conformity norms rather than individual competence perceptions (Hofstede, 2001). This contextual influence may explain why SE failed to moderate the TL–LE pathway. In contrast, socio-emotional competence, which is more relationally embedded and responsive to interpersonal cues, may depend more on sustained relational and experiential inputs than on short-term motivational boosts from leadership behaviors (Bandura, 1997; Jennings and Greenberg, 2009; Wentzel, 2010). The differential moderating role of SE between relational variables (e.g., TSR) and behavioral engagement (LE) underscores potential construct alignment issues, where LE—as a task and behavior-oriented construct—is driven more strongly by situational and environmental factors than by individual self-efficacy alone.

In sum, by integrating transformational leadership theory, the SEL framework, and social cognitive theory, the present study corroborates established mechanisms while highlighting nuanced conditional effects. These findings emphasize the importance of relational and motivational pathways in fostering socio-emotional development in higher education and underscore the need for future research to investigate boundary conditions. Specifically, incorporating multi-level contextual variables and longitudinal designs could elucidate the dynamic interplay between leadership, efficacy beliefs, engagement, and SEC, thereby refining theoretical models and informing targeted leadership interventions.

### 5.6 Theoretical implications

This study advances the literature on educational leadership by clarifying the specific psychological pathways through which TL fosters university students’ SEC. By identifying TSR and LE as sequential and parallel mediators, the findings demonstrate that TL exerts its strongest socio-emotional impact through the relational pathway (via TSR), while the task-oriented pathway (via LE) plays a complementary but less self-efficacy–dependent role. Furthermore, the moderating role of SE on the TL–TSR link underscores that students’ competence beliefs amplify the relational benefits of TL but do not similarly strengthen task engagement. This nuanced pattern enriches transformational leadership theory by integrating relational embeddedness with self-efficacy theory (Bandura, 1997), suggesting that leadership behaviors influence socio-emotional development most effectively when they activate both interpersonal trust and motivational self-beliefs.

### 5.7 Practical implications

The findings offer concrete, evidence-based strategies for higher education institutions to enhance students’ socio-emotional development through targeted leadership practices and support systems.

#### 5.7.1 Cultivating transformational leadership behaviors aligned with mediators

Faculty development programs should train instructors in TL behaviors that directly activate TSR and LE. For the TSR pathway, emphasize empathy, trust-building dialogues, and individualized mentoring. For the LE pathway, focus on stimulating intellectual challenge, goal clarity, and scaffolding that fosters persistence.

#### 5.7.2 Leveraging self-efficacy in relational contexts

Since SE strengthens the TL–TSR link, universities can integrate self-efficacy–building techniques—such as structured mastery experiences, constructive feedback, and vicarious modeling—into relationship-rich activities (e.g., research supervision, project-based learning) to maximize socio-emotional gains.

#### 5.7.3 Embedding active learning to sustain engagement

While LE is less contingent on SE in this study’s context, its mediating role indicates that engagement-focused pedagogies remain crucial. Faculty can adopt cooperative learning structures, problem-based tasks, and reflective exercises to foster behavioral, emotional, and cognitive involvement.

#### 5.7.4 Creating culturally responsive support systems

In collectivist educational settings, relationship-centered interventions may yield stronger socio-emotional outcomes than purely task-oriented approaches. Policies should therefore prioritize faculty–student rapport and collaborative academic communities alongside rigorous curriculum design.

By systematically aligning leadership behaviors with the identified mediators (TSR, LE) and conditional mechanisms (SE), universities can design emotionally supportive and intellectually stimulating environments that actively enhance students’ social–emotional competence, rather than relying on general leadership development initiatives.

### 5.8 Limitations and future directions

While providing valuable insights, the research contains certain limitations requiring acknowledgment. Firstly, due to its cross-sectional design, this research provides correlational rather than causal evidence regarding the relationships among TL, TSR, LE, and social–emotional competence. While structural equation modeling techniques enhance interpretative clarity, future studies employing longitudinal or experimental designs would provide stronger causal validation of these pathways.

Secondly, all measures utilized self-report questionnaires, which might introduce common method bias and subjective response tendencies. To mitigate this, subsequent research should incorporate multi-informant methods (e.g., teacher ratings, peer evaluations) and objective behavioral measures of social–emotional skills, engagement, and teacher-student interactions, thereby enhancing data reliability and reducing bias.

Thirdly, the generalizability of these findings, while robust within the sampled context (college students from diverse Chinese universities), remains limited regarding international applicability. Cross-cultural replication studies would further clarify how varying educational and cultural contexts affect the applicability and robustness of the proposed mediation and moderation models. Moreover, considering potential institutional variability, future research should explicitly investigate differences across academic disciplines (e.g., STEM vs. humanities/social sciences) and institutional characteristics (e.g., public vs. private universities).

Finally, although this study highlights self-efficacy as a critical moderator, future research could explore additional psychological variables—such as emotional intelligence, resilience, or personality traits—as potential moderators or mediators. Incorporating these variables would deepen understanding of how individual differences influence students’ responsiveness to leadership and educational environments.

## 6 Conclusion

This study offers new empirical insights into the associations between university teachers’ TL and students’ SEC, with TSR and LE serving as potential mediating mechanisms, and SE as a moderating factor. The findings suggest that TL is not only directly related to students’ SEC but also indirectly associated through enhanced relational bonds and greater learning engagement—both of which are important elements in students’ social and emotional functioning.

Furthermore, self-efficacy appears to play a moderating role in this association. In alignment with Bandura’s self-efficacy theory, students with higher SE may be more likely to benefit from transformational teaching behaviors and supportive learning environments. These students might perceive such environments as more empowering, thereby experiencing higher levels of SEC. While the cross-sectional nature of the data precludes causal conclusions, the observed patterns are consistent with theoretical propositions suggesting a synergistic relationship between leadership, learner characteristics, and socio-emotional outcomes.

From a theoretical perspective, this study contributes to a more integrative understanding of how external instructional factors and internal learner traits may interact to support students’ social–emotional development in higher education. From a practical standpoint, the results underline the importance of fostering transformational teaching practices alongside efforts to cultivate student self-efficacy. Higher education institutions are encouraged to provide professional development opportunities that support teachers in adopting leadership behaviors aligned with transformational principles, while also implementing strategies that enhance students’ confidence and motivation in academic contexts.

Future research would benefit from using longitudinal or experimental designs to examine the temporal and directional nature of these associations. Replicating this model across diverse institutional and cultural contexts could further clarify the mechanisms through which leadership and personal agency contribute to students’ socio-emotional growth.

## Data Availability

The raw data supporting the conclusions of this article will be made available by the authors, without undue reservation.
